# Independently founded populations of *Sclerotinia sclerotiorum* from a tropical and a temperate region have similar genetic structure

**DOI:** 10.1371/journal.pone.0173915

**Published:** 2017-03-15

**Authors:** Miller S. Lehner, Trazilbo J. de Paula Júnior, Emerson M. Del Ponte, Eduardo S. G. Mizubuti, Sarah J. Pethybridge

**Affiliations:** 1 School of Integrative Plant Science, Plant Pathology & Plant-Microbe Biology Section, Cornell University, Geneva, New York, United States of America; 2 Empresa de Pesquisa Agropecuária de Minas Gerais (EPAMIG), Viçosa, Minas Gerais, Brazil; 3 Departamento de Fitopatologia, Universidade Federal de Viçosa, Viçosa, Minas Gerais, Brazil; National Cheng Kung University, TAIWAN

## Abstract

*Sclerotinia sclerotiorum* populations from tropical agricultural zones have been suggested to be more variable compared to those from temperate zones. However, no data were available comparing populations from both zones using the same set of markers. In this study, we compared *S*. *sclerotiorum* populations from the United States of America (USA, temperate) and southeast Brazil (tropical) using the frequency of mycelial compatibility groups (MCGs) and 13 microsatellite (SSR) markers. Populations were sourced from diseased plants within leguminous crops in New York, USA (NY; *n* = 78 isolates), and Minas Gerais State, Brazil (MG; *n* = 109). Twenty MCGs were identified in NY and 14 were previously reported in MG. The effective number of genotypes based on Hill’s number of order 0, which corresponded to the number of multilocus genotypes (MLGs) were 22 (95% CI = 15.6–28.4) and 24 (95% CI = 18.9–29.1) in NY and MG, respectively. Clonal fractions of MLGs were 71.8% (NY) and 78.0% (MG). The effective number of genotypes based on Hill’s number of orders 1 and 2 in NY were 8.9 (95% CI = 5.2–12.6) and 4.4 (95% CI = 2.6–6.1), respectively. For MG these indices were 11.4 (95% CI = 8.7–14.1) and 7.1 (95% CI = 5.1–9.0), respectively. There were no significant differences of allelic richness, private allelic richness, gene diversity, effective number of alleles and genotype evenness between the NY and MG populations. The populations were differentiated, with 29% of total variance attributed to differences between them and G''_ST_ and Jost’s D indices higher than 0.50. Cluster analysis revealed dissimilarity higher than 80% among most MLGs from both populations. Different alleles segregated in the populations but both had similar levels of genotypic variability.

## Introduction

*Sclerotinia sclerotiorum* is amongst the most devastating and recalcitrant plant pathogens that infect a broad range of hosts and may lead to substantial crop losses [1–3]. Genetic variability of the pathogen has been investigated in many countries using different sets of molecular markers during the last 25 years [4]. Earlier studies used restriction fragment length polymorphism (RFLP) and/or mycelial compatibility groups (MCGs) to estimate the variability within the populations. Most of the RFLP-based studies depicted a clonal genetic structure in North American populations from Canada [5–8] or from North Carolina [9] and Washington State [10] in the United States of America (USA). These findings are consistent with the homothallic nature of the pathogen that reproduces predominantly by self-fertilization or by production of somatic resting structures called sclerotia [11].

More recently, simple sequence repeats (SSR, or microsatellites) markers have also been used to assess the genetic variability in *S*. *sclerotiorum* populations. While some studies have reported high variability and outcrossing signals [12–15], others have depicted a clonal genetic structure of the pathogen population [16–18]. The factors contributing to the variability have been investigated and an association with climate has been speculated [19]. However, thus far, this speculation has been done by studies that used populations from disparate temperate or tropical regions, with no direct comparison. In addition, the use of different sets of genetic markers in the published studies masks reliable comparisons.

In the majority (80%) of genetic variability studies of the *S*. *sclerotiorum* populations from subtropical or tropical regions, the levels of genetic diversity were reported to be high, with most (63%) of them using SSR markers. In temperate regions, a clonal genetic structure was reported in 63% of the studies using mainly (44% of the studies) RFLP markers [4]. As Lehner and Mizubuti [4] argue, the potential effect of climatic zones on the genetic diversity may be confounded with the differential resolution of distinct molecular markers. Therefore, a direct comparison of *S*. *sclerotiorum* populations from distinct climatic zones using the same set of molecular markers may help to address this question.

In the tropical climatic zone, as the case of southeast Brazil, many susceptible crops can be grown in succession year-round, providing an extended period over which plants may be infected by *S*. *sclerotiorum*. This "green bridge" effect can lead to a higher number of generations of the pathogen and, consequently, higher chance for generation, recombination and maintenance of genetic variability in the population than in temperate regions, where a long and harsh winter prevents the growth of multiple crops year-round. Genetic variability studies comparing *S*. *sclerotiorum* populations from distinct climates zones were conducted with subtropical and temperate populations within the USA using DNA sequence data [19], and between China and the USA using SSR markers [13]. Carbone and Kohn [19] reported higher variability in subtropical populations than in those from the temperate region within the USA. Using SSR markers, populations from a subtropical region in mainland China exhibited similar gene diversity, but higher genotypic diversity than those from temperate regions within the USA [13].

In addition to the indirect comparisons using inconsistent sets of markers, there have also been analytical limitations when comparing the diversity of *S*. *sclerotiorum*. In only a few cases the genetic structure, i.e. the amount and distribution of genetic variability in the populations, was robustly addressed. Usually, only the amount of genetic variability is compared. Additionally, most studies in plant pathology make use of species diversity indices, mainly Shannon-Wiener H’ and Simpson’s diversity (1/S) or a variant of it, the Stoddart and Taylor’s G, as proxies for pathogen genotype diversity [20]. It has been demonstrated that these indices do not reflect true diversity values and do not allow for direct assessment of the assemblages [21]. Thus, to allow for proper comparisons of diversity it is better to use the effective number of genotypes or Hill’s number [22]. The effective number of genotypes (Hill’s number) is defined as the number of equally abundant genotypes required to reflect the value of a diversity measure [21]. Even though Hill’s number provides a better estimate of true diversity it can be misleading when sample sizes [20] or sample-completeness vary [21]. Sample completeness is estimated by coverage, which is defined as “the total relative abundances of the observed species, or equivalently, the proportion of the total number of individuals in an assemblage that belong to species represented in the sample” [21]. Thus, when comparing different samples that vary in size or coverage, one should make comparisons based on standardized sample size either smaller or larger, or standardized sample completeness (coverage). Recently, new analytical approaches were developed that allow for comparisons of the effective number of genotypes from a sample either rarefied or extrapolated when compared to smaller or larger samples (sample-size based comparison), respectively [23]. Similarly, those tools also allow for comparisons of the effective number of genotypes when sample completeness vary (coverage-based comparison) [24]. Now, using the same set of markers and proper analytical tools for comparisons, the objective of this study was to compare the diversity of two populations of *S*. *sclerotiorum*: one from a temperate zone in the USA and another from a tropical zone in Brazil using SSR markers and frequency of MCGs data.

## Materials and methods

### *Sclerotinia sclerotiorum* isolates

DNA of 109 *S*. *sclerotiorum* isolates collected from 20 dry bean (*Phaseolus vulgaris* L.) fields in Minas Gerais State (MG), Southeast Brazil, were supplied by the Universidade Federal de Viçosa. This area is classified as a tropical climatic zone given its localization between the tropics of Cancer and Capricorn. The isolates from the temperate zone (≥ 40° latitude) were collected across western and central New York State (NY), USA. Seventy-eight isolates were collected from dry bean (*n* = 8 fields), snap bean (*P*. *vulgaris; n* = 4), lima bean (*P*. *lunatus; n* = 1) and soybean (*Glycine max; n* = 3). The number of isolates collected within each field varied from 2–12 in Brazil, and 3–6 in the USA. In both regions, the distance among sampling locations within the fields was at least 10 m. Colonies obtained following myceliogenic germination of sclerotia on potato-dextrose agar and hyphal-tip isolations were conducted as described by Lehner et al. [18].

### DNA extraction and *S*. *sclerotiorum*-specific PCR

DNA of *S*. *sclerotiorum* isolates from Brazil was extracted as described by Lehner et al. [18]. The DNA of isolates from the USA was extracted using the Wizard Genomic DNA Purification Kit (Promega, Madison, WI, USA) following the manufacturer’s instructions with the following modification: after the centrifugation using the "Protein Precipitation Solution" the supernatant was transferred to a clean microcentrifuge tube containing 300 μl of chloroform- isoamyl alcohol (24:1) and 300 μl of 2% solution of alkyltrimethylammonium bromide (Sigma-Aldrich, St. Louis, MO, USA). The tubes were centrifuged for 10 minutes at 14,000 rpm and the supernatant was collected. The remainder of the DNA extraction was performed according to the manufacturer’s instructions. The integrity of the DNA samples was analyzed using electrophoresis on a 1% agarose gel (1% wt/vol agarose in Tris-borate-EDTA [TBE]) amended with 0.5× (v/v) nucleic acid stain GelRed (Biotium, Inc., Hayward, CA). The DNA concentration for each isolate was adjusted to 30 ng/μl.

The identity of each isolate was confirmed using the primer pair SscadF1/SscadR1 specific for *S*. *sclerotiorum* [25]. DNA of the *S*. *sclerotiorum* ‘1980’ isolate obtained from Dr. J. Steadman, University of Nebraska, NE, USA was used as a positive control. Water and DNA of two isolates of *S*. *trifoliorum* (06CWM-G22, L-type and 06CWM-G27, S-type) previously characterized by Njambere et al. [26] were used as negative controls. PCR reactions were performed in a final volume of 20 μl with 2 μl of DNA, 0.5 μl of each primer at 10 mM, 0.5 μl of dNTPs at 10 mM, 2 μl of the 10x Standard Taq Reaction Buffer that includes 1.5 mM MgCl_2_, 10mM Tris-HCl and 50 mM KCl (New England Biolabs Inc., Ipswich, MA, USA) and 0.1 μl of Taq DNA polymerase (New England Biolabs, Inc.). PCR reactions were conducted in a C1000 Thermal Cycler (Bio-Rad, Hercules, CA, USA). PCR conditions were an initial denaturation for 5 minutes at 95°C, followed by 35 cycles of denaturation at 95°C for 30 seconds, annealing at 57°C for 30 seconds, and extension at 68°C for 30 seconds; and a final extension at 68°C for 5 minutes. Amplification was confirmed by using electrophoresis in a 1% agarose gel and TBE and viewed under UV light after staining with GelRed (Biotium, Hayward, CA, USA). The size of fragments was estimated by comparison to a 100 bp DNA ladder and species were identified based on comparison to *S*. *sclerotiorum* and *S*. *trifoliorum* controls [25].

#### Mycelial compatibility groupings

The MCGs of MG isolates were previously reported by Lehner et al. [18]. The MCGs of NY isolates were determined using the same method. The MCGs were determined for each population separately. There were no pairings between MG and NY isolates. In brief, pairing of the isolates was performed on PDA supplemented with 75 μl/L of McCormick’s red food coloring [27]. Four pairings were performed at equidistant intervals within each 60 mm diameter Petri plate. Plates were maintained at 23°C in the dark. Mycelial compatibility was assessed after 3 and 6 days of incubation. Each pairing was performed twice. When the results were inconsistent, two new independent pairings were conducted.

#### Genotyping

Primer pairs flanking 13 SSR loci as described by Sirjusingh and Kohn [28] were used to quantify genetic variability. The forward primers were labeled with the fluorescent dyes 6-FAM, VIC, NED or PET (Table 1). Multiplex PCR reactions were performed using the Multiplex PCR 5X Master Mix kit as described by the manufacturer (New England Biolabs, Inc.). DNA of the *S*. *sclerotiorum* ‘1980’ isolate was used as positive control [29]. For ten isolates, two independent DNA extractions and genotyping reactions were performed to confirm reproducibility. PCR products were diluted 1:50 and analyzed using the GeneScan-500 LIZ size standard (Applied Biosystems) on an ABI 3730xl DNA analyzer at the Cornell University Institute of Biotechnology Genomic Diversity Facility, Ithaca, NY, USA. Fragment analysis was performed using the software GENEMARKER v.1.191 (SoftGenetics). The size of DNA fragments were manually binned into alleles according to the number of repeat units at each locus.

**Table 1:** Number of alleles and genetic differentiation per locus between the *Sclerotinia sclerotiorum* populations from New York (NY), USA and Minas Gerais State (MG), Brazil. Estimates were obtained for 13 SSR loci located in eight distinct chromosomes.

Locus (Chromosome)	Repetitive sequence	Dye	Number of alleles		G''_ST_	D
			NY	MG		
55–4 (15)	(TACA)_10_	NED	4	5	0.98	0.95
13–2 (6)	(GTGGT)_6_	6-FAM	3	5	0.97	0.94
110–4 (6)	(TATG)_9_	PET	3	2	0.92	0.86
8–3 (11)	(CA)_12_	NED	4	6	0.13	0.07
5–2 (3)	(GT)_8_	6-FAM	2	3	0.73	0.51
17–3 (4)	(TTA)_9_	VIC	3	7	0.86	0.74
114–4 (4)	(AGAT)_14_(AAGC)_4_	PET	5	9	0.95	0.93
7–2 (4)	(GA)_14_	VIC	3	5	0.97	0.39
12–2 (5)	(CA)_9_	PET	3	4	0.37	0.26
9–2 (6)	(CA)_9_(CT)_9_	NED	2	3	0.00	0.00
92–4 (1)	(CT)_12_	6-FAM	3	4	0.71	0.57
36–4 (6)	(CA)_6_(CGCA)_2_(CAT)_2_	6-FAM	1	1	-	-
42–4 (8)	(GA)_9_	VIC	1	1	-	-

G''_ST_ index [41]; Jost’s D index [42]

To validate the size scoring obtained in the fragment analysis, each SSR allele was sequenced using a representative isolate. Uniplex PCR reactions were performed using non-fluorescent primers as previously described for the species-specific assay. Excess nucleotides were removed prior to sequencing using ExoSAP-IT according to the manufacturer’s instructions (Affymetrix, Cleveland, OH, USA) and sequenced at the Cornell University Institute of Biotechnology Genomic Diversity Facility. The nucleotide sequences were edited using DNA BASER sequence assembly software (Heracle BioSoft) and aligned using MEGA 5.0 [30]. To validate the SSR alleles we included in the alignments the sequences of the isolate LMK 211 [28]. The number of repeated sequences of each allele was compared to the reference sequences of the isolate LMK 211.

### Data analysis

#### Genetic diversity and linkage disequilibrium

Allelic richness (AR) and private allelic richness (pAR) were estimated after rarefaction for the smallest sample size using HP-RARE [31]. The effective number of alleles (Ne) in each population was calculated using GENODIVE [32]. Gene diversity [33] was calculated for each population using the *poppr* package for R [34].

Multilocus genotypes (MLGs) were constructed for each isolate combining the alleles identified at each SSR locus, excluding monomorphic loci. The clonal fraction in each population was calculated as 1 - [(number of different genotypes) / (total number of isolates)] [35]. Based on the frequency of MLGs in each population, true diversity estimated by Hill’s numbers or the effective numbers of genotypes were calculated. Hill’s numbers (N) of orders 0, 1, and 2 were calculated for each population [36]. Accordingly, Hill’s number N0, N1 or N2 correspond to estimates of genotype richness, the exponential of Shannon’s entropy, and the inverse of the Simpson’s concentration indices, respectively [21]. Integrated curves that allow rarefaction and extrapolation were used to compare these numbers from samples of different sizes using the non-asymptotic approach [23]. For each curve, the 95% confidence interval (95% CI) was constructed and plotted. Diversity in the different populations was compared based on the overlapping of the 95% CIs. Similar analyses were conducted assuming variation in sample completeness. In this case, samples were standardized according to coverage (coverage-based) and Hill’s numbers were calculated using the non-asymptotic approach [24]. The diversity analyses were conducted using the iNEXT package [37] for R.

In addition to genotypic diversity, the modified Hill’s ratio E5 evenness index was also calculated [38]. Evenness index represents the relative abundance of the genotypes in a sample. Thus, this index is high when all genotypes occur with similar frequencies and low when few genotypes predominate while others occur at low frequency. The E5 index was calculated using the *poppr* package [34].

Genotype accumulation curves were produced for each population using 1,000 resamplings. The *r*_*D*_ index was calculated from clone-corrected data set using 999 permutations to estimate the linkage disequilibrium across the SSR loci. Linkage disequilibrium between all pairs of locus was also tested using MULTILOCUS program [39] and 1,000 randomizations of the clone-corrected data set. The accumulation curves and the *r*_*D*_ index were also calculated using the *poppr* package [34].

#### Genetic differentiation

The partitioning of variation between and within the populations was assessed by a hierarchical analysis of molecular variance (AMOVA) from the clone-corrected data set using the Arlequin program [40]. The distance method was the sum of squared size differences between two MLGs using 1,000 permutations. The GENODIVE program [32] was used to calculate the G''_ST_ [41] and the Jost’s D [42] indices per locus and averaged over all loci, as measures of population differentiation. Both, G''_ST_ and Jost’s D range from zero (no differentiation) to one (complete differentiation). These indices were selected because they use different principles to measure genetic differentiation. G''_ST_ index varies with the diversity within populations, while Jost’s D is based on the effective number of alleles [41]. A dissimilarity matrix reflecting the percentage of allelic differences (dissimilarity) among the MLGs was calculated followed by a hierarchical cluster analysis according to the complete linkage method. Clusters were assigned for MLGs with at least 50% of similarity. The *poppr* package [34] was used to calculate the dissimilarity matrix.

## Results

### Species-specific PCR and mycelial compatibility groupings

**Fig 1:**
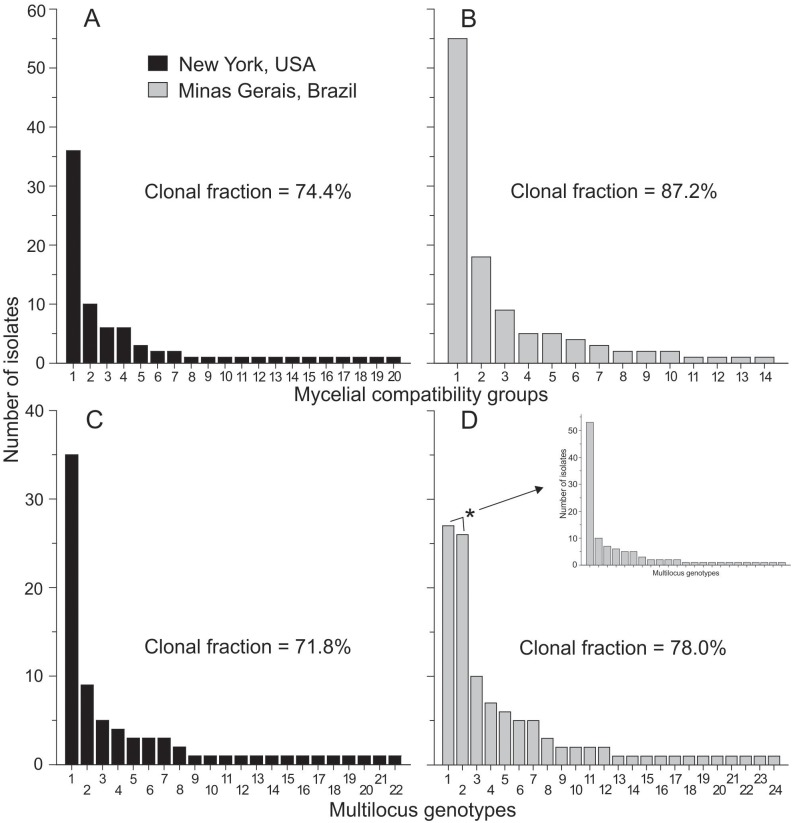
Frequency of the mycelial compatibility groupings (A and B) and multilocus genotypes (MLGs) (C and D) obtained from 13 microsatellite loci in the *Sclerotinia sclerotiorum* populations from New York, USA, and Minas Gerais, Brazil. The asterisk indicates the sum of the frequencies of two closely related MLGs, with a difference at the 8–3 locus (dinucleotide repeats). The MLG 1 has a 250 base pair (bp) allele while the MLG 2 has a 252 bp allele.

The identity of all isolates was confirmed by the amplification of the expected 100 bp-fragment of the calmodulin gene. The DNA of the *S*. *trifoliorum* isolates was not amplified (S1 Fig). Twenty MCGs were identified among the isolates from NY (Fig 1A). In MG, 14 MCGs were previously identified among the 109 isolates (Fig 1B). The number of isolates within each MCG ranged from 1–36 in NY and 1–55 in MG, corresponding to clonal fractions of 74.4% and 87.2%, respectively (S1 Table).

### Genotyping

#### Validation of alleles

Allele sizes from each of the isolates used in the validation test were reproducible at all SSR loci. The allele sizes identified using DNA of the *S*. *sclerotiorum* ‘1980’ reference isolate were also confirmed. Moreover, the size of each SSR locus sequence was in agreement with the binning of results obtained in the fragment analysis (S1 Table).

#### Genetic diversity and linkage disequilibrium

Loci 36–4 and 42–4 were monomorphic in both populations. In NY, the number of alleles ranged from two (locus 5–2 and 9–2) to five (locus 114–4). In MG, the number of alleles ranged from two (locus 110–4) to nine (locus 114–4) (Table 1). There was no significant difference in AR and pAR between the two populations. Gene diversity and the Ne were also not significantly different between NY and MG (Table 2).

**Table 2:** Population genetic parameters of *Sclerotinia sclerotiorum* populations from New York (NY), USA and Minas Gerais State (MG), Brazil. Sd. = standard deviation and 95% CI = 95% confidence interval.

Parameters	Populations	
	NY	MG
Sample size	78	109
Allelic richness after rarefaction (n = 78) ± sd	3.08 ± 0.79	4.51 ± 1.75
Private allelic richness after rarefaction (n = 78) ± sd	0.94 ± 1.03	2.37 ± 1.49
Gene diversity [33] ± sd	0.61 ± 0.16	0.66 ± 0.08
Effective number of alleles (95% CI)	1.86 (1.57–2.18)	2.18 (1.84–2.51)
Evenness as E_5_ index (95% CI)	0.42 (0.33–0.53)	0.58 (0.51–0.66)
r_D_ (multilocus linkage disequilibrium from clone-corrected data) (P-value)	0.12 (*P* = 0.001)	0.14 (*P* = 0.001)

**Fig 2:**
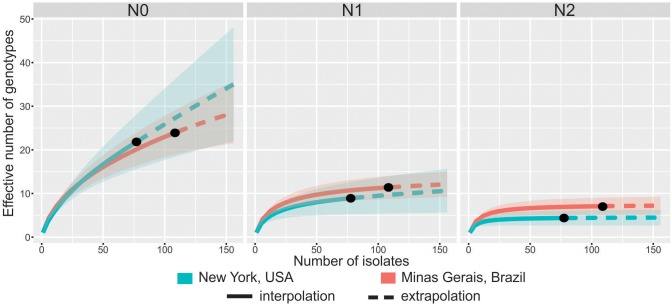
Diversity accumulation curves for different sample sizes with 95% confidence intervals (shaded areas) of the Hill’s numbers or effective number of genotypes of orders 0 (N0), 1 (N1) and 2 (N2) estimated for the *Sclerotinia sclerotiorum* populations from New York, USA and Minas Gerais, Brazil. The N0, N1 and N2 numbers correspond to genotype richness, the exponential of Shannon’s entropy, and the inverse of the Simpson’s concentration indices, respectively. Solid lines correspond to rarefaction (interpolation) and dashed lines to extrapolation curves up to the base sample size of 156 individuals which corresponds to double the smaller reference sample size (NY = 78). The 95% confidence intervals were obtained by a bootstrap method based on 200 replications.

The genotypic richness assessed by Hill’s number of order 0 (N0) of both populations was similar (Fig 2A). In NY, 22 (95% CI = 15.6–28.4) MLGs were identified among the 78 isolates. In MG, 24 (95% CI = 18.9–29.1) MLGs were identified among the 109 isolates. Clonal fractions of MLGs were 71.8% and 78.0% in NY and MG, respectively. The effective number of genotypes based on Hill’s number of order 1 (N1 index) was 8.9 (95% CI = 5.2–12.6) and 11.4 (95% CI = 8.7–14.1) for NY and MG, respectively. The N2 index was 4.4 (95% CI = 2.6–6.1) and 7.1 (5.1–9.0) for NY and MG, respectively. Therefore, no significant difference in effective number of genotypes based on Hill’s numbers was observed between NY and MG (Fig 2). Similar trends were observed when samples were standardized by coverage (S1 Fig).

**Fig 3:**
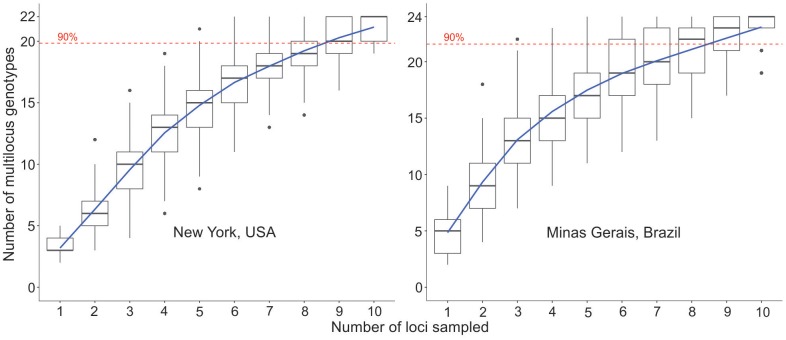
Genotype accumulation curves for the *Sclerotinia sclerotiorum* populations from New York, United States (USA) and Minas Gerais, Brazil. The number of loci was randomly sampled (1,000 times) without replacement up to n − 1 loci. The 90% of the number of multilocus genotypes identified in each population are indicated by dashed lines.

No MLG was shared between NY and MG. The most frequent MLG in the NY population was detected in 35 isolates (Fig 1C). In MG, the most frequent MLGs were identified in 27 and 26 isolates (Fig 1D). Evenness did not differ significantly between the two populations (Table 2). The genotype accumulation curves indicated that in both populations, 90% of the MLGs were detected with eight or nine SSR markers (Fig 3). However, a typical plateau in the number of MLGs was not observed for both populations.

The overall r_D_ index in both populations was significantly different from zero (Table 2). However, the pairwise test revealed that 60% of pairs of loci in the NY population were at linkage equilibrium (*P* > 0.05), while in MG, this proportion was equal to 45% (data not shown).

#### Genetic differentiation

**Fig 4:**
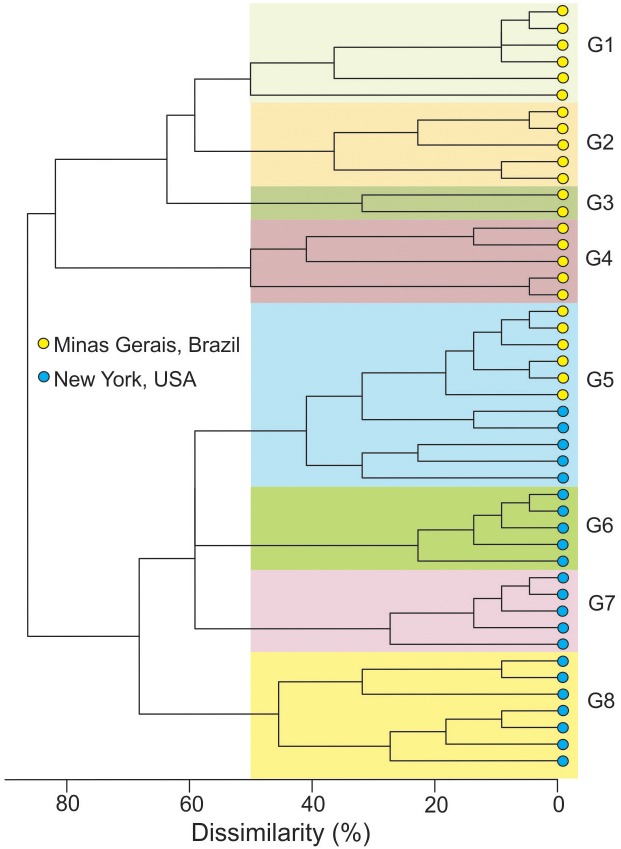
Hierarchical cluster analysis according to complete linkage method from a dissimilarity matrix reflecting the percentage of allelic differences among the Multilocus Genotypes (MLGs). Clusters (G) were assigned for MLGs with at least 50% of similarity.

The G''_ST_ index depicted a high level of genetic differentiation (G''_ST_ = 0.75) and ranged from zero (locus 9–2) to 0.98 (locus 55–4). This was corroborated by the magnitude of the Jost’s measure of differentiation (D = 0.58), which ranged from zero (locus 9–2) to 0.95 (locus 55–4) (Table 1). AMOVA identified that the variation between the populations represented 29.1% of total variance, while 70.9% was attributed to variation within populations. There was high dissimilarity (> 80%) when most of the MLGs from each population were paired. Based on 50% similarity, eight clusters were identified. Seven of these clusters were comprised of isolates solely from MG or NY. However, group 5 was comprised of MLGs from MG and NY with similarity of at least 50% (Fig 4).

## Discussion

*Sclerotinia sclerotiorum* populations from a tropical climatic zone (MG, Brazil) and a temperate zone (NY, USA) exhibited similar level of genetic diversity when two markers were used: frequency of MCGs and SSR markers. Despite the larger sample size of the MG population, the number of MCGs was smaller than in the NY population. Consequently, the clonal fraction in MG (87.2%) was higher than in the NY population (74.4%). This finding is consistent with earlier estimates of MCG diversity in *S*. *sclerotiorum* populations. Clonal fractions of MCGs in previous studies from temperate regions ranged from 34.4% [17] to 90.1% [8], with an average of 53.6% across six studies [5,8,12,13,15,17]. Information on MCG diversity in tropical zones is available from a study conducted in Brazil, which reported 72% of clonal fraction [43]. Direct comparisons are somewhat limited because there are six times more studies conducted with populations from temperate than from tropical climatic zone. Collectively, results of the current and previous studies with populations from a single climatic zone do not support the hypothesis of higher MCG diversity associated with *S*. *sclerotiorum* from tropical zones.

The distribution of MCGs frequencies in the two populations were similar, but were slightly different for the MLGs. In NY, only one MLG was present at high frequency (> 25 isolates). In MG, two MLGs (MLG1 and 2) in high frequency were identified. We reconstructed the MLGs histogram in MG summing the frequency of MLGs 1 and 2, which resulted in a similar frequency to that observed in NY (Fig 1D inset). The MLGs 1 and 2 from MG belong to the same MCG. They exhibited differences only at the 8–3 SSR locus which consists of dinucleotide repeats. The MLG1 and MLG2 have allele sizes of 250 and 252 bp, respectively. Variation at one or few SSR loci among isolates belonging to the same MCG has also been reported in other studies [17,44]. These slight variations probably are not related to pathogenicity/virulence and isolates can be considered to be genetically and physiologically almost identical.

Higher variability in *S*. *sclerotiorum* populations from tropical zones could be linked to the higher number of generations of the pathogen associated with the availability of host crops throughout the year and consequently higher genetic variability. In the present study, both populations exhibited at least 40% of pairs of loci in linkage equilibrium. Moreover, in both populations there was a decoupling between MCGs and SSR markers. In the NY population, six MLGs were associated with more than one MCG, while in MG this occurred for one MLG. Linkage equilibrium between pairs of loci and unlinking of markers suggest the involvement of some recombination process [45] in both populations. The SSR markers characterize a small portion of the *S*. *sclerotiorum* genome. Therefore, a further study using higher-resolution genomic tools or the use of additional markers may provide further insight into the differential contribution of recombination in *S*. *sclerotiorum* populations from distinct climatic zones.

The *S*. *sclerotiorum* populations from NY and MG were highly differentiated (G''_ST_ or Jost’s D > 0.5) at eight of the 13 SSR loci. Populations from widely separated areas, a subtropical region of China (*n* = 30) and a temperate region of the USA (*n* = 29), were also highly differentiated at SSR loci [13]. Large geographic distances and absence of human-mediated ways allowing gene flow between these regions explain such differentiation. These arguments can also apply to the present study, since NY and MG did not share MLGs. However, a careful analysis of the allelic profile of some MLGs from NY and MG revealed some that were genetically similar, with dissimilarity lower than 50%. These MLGs from MG belonged to MCG2, while those from NY belonged to distinct MCGs. These isolates may therefore be related by a distant common parent. Nevertheless, a more accurate analysis with DNA sequences, using phylogeny and simulations of genealogies using the coalescent approach could clarify this question.

*Sclerotinia sclerotiorum* populations from tropical and temperate zones exhibited substantial (> 70%) clonality. Several studies from different geographic regions have reported clonality in *S*. *sclerotiorum* populations [5,8,9,17,18]. However, some studies identified high levels of genotypic diversity associated with evidence of outcrossing, such as linkage equilibrium and sibling ascospores being genetically distinct [12,14,44,46]. Differences in the estimates of diversity among the studies are expected. However, there are still insufficient data to answer why some *S*. *sclerotiorum* populations tend to be panmictic (random mating). This has important implications for the development of management strategies, because pathogen populations in which random mating takes place tends to evolve faster [45]. Consequently, the probability of the pathogen overcoming host resistance or developing resistance to fungicides becomes higher [45]. Here, there was no relationship between the climate of origin with the amount of diversity or with evidences of outcrossing in the populations. Furthermore, the populations investigated probably resulted from independent founder events but evolved in a similar way. They both maintain a clonal structure with the predominance of a few lineages that seem to be widely distributed and well adapted to cause white molds in leguminous crops.

## Supporting information

S1 Fig Electrophoresis gel photo of PCR products obtained in the species-specific PCR assay to assess identity of the isolates from New York (NY, USA) and Minas Gerais (MG, Brazil). The amplification of DNA fragments of 100 base pairs (bp) is specific to *Sclerotinia sclerotiorum* isolates.
(TIF)

S2 Fig Diversity accumulation curves for different sample coverage (an estimate of sample completeness) with 95% confidence intervals (shaded areas) of the Hill’s numbers or effective number of genotypes of orders 0 (N0), 1 (N1) and 2 (N2) estimated for the *Sclerotinia sclerotiorum* populations from New York, USA and Minas Gerais, Brazil. The N0, N1 and N2 numbers correspond to genotype richness, the exponential of Shannon’s entropy, and the inverse of the Simpson’s concentration indices, respectively. Solid lines correspond to rarefaction (interpolation) and dashed lines to extrapolation curves. The 95% confidence intervals were obtained by a bootstrap method based on 200 replications.
(TIF)

S1 Table Mycelial Compatibility Group (MCG) and allele size at each microsatellite locus of the *Sclerotinia sclerotiorum* isolates from New York, United States of America (USA), and Minas Gerais, Brazil (BRA). (DOCX)
