# Supplementary figures and images for: Independently founded populations of Sclerotinia sclerotiorum from a tropical and a temperate region have similar genetic structure
Source: PLoS One. 2017 Mar 15;12(3):e0173915. doi: 10.1371/journal.pone.0173915 (PMC5352009; doi:10.1371/journal.pone.0173915)

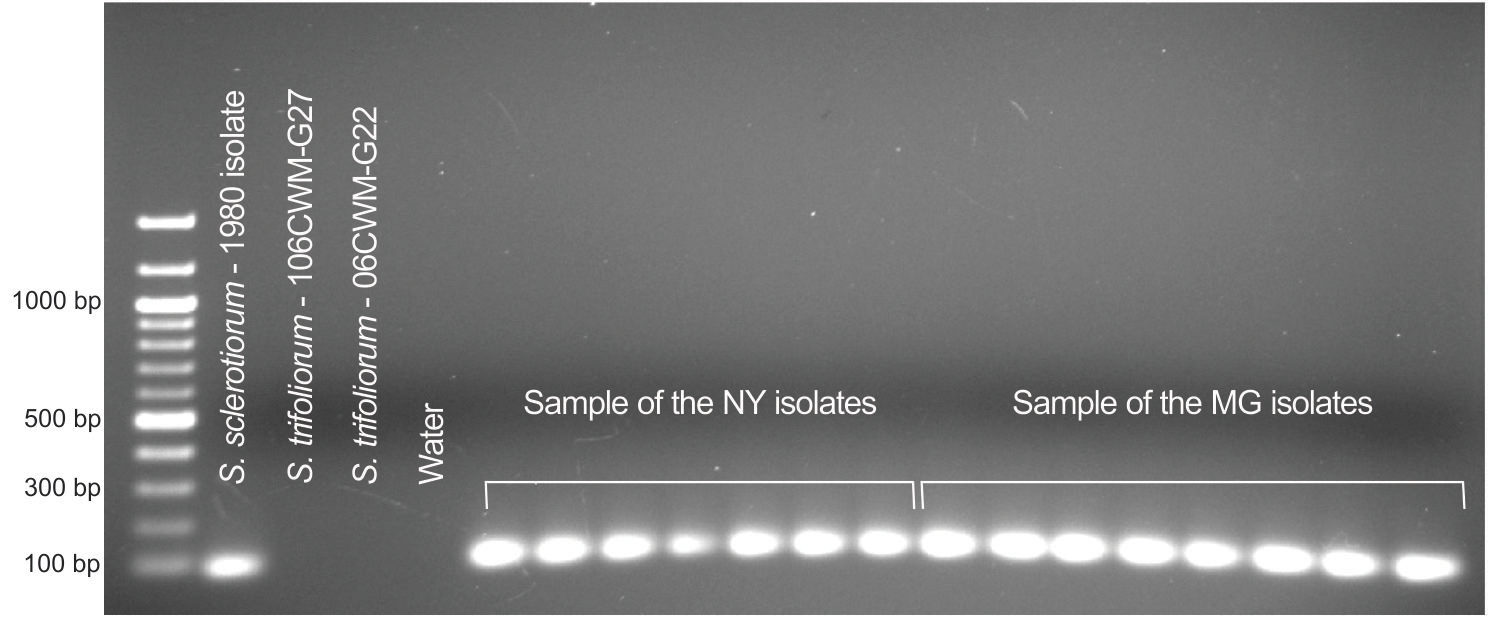

Supplement: S1 Fig — The amplification of DNA fragments of 100 base pairs (bp) is specific to Sclerotinia sclerotiorum isolates. (TIF) [file pone.0173915.s001.tif]

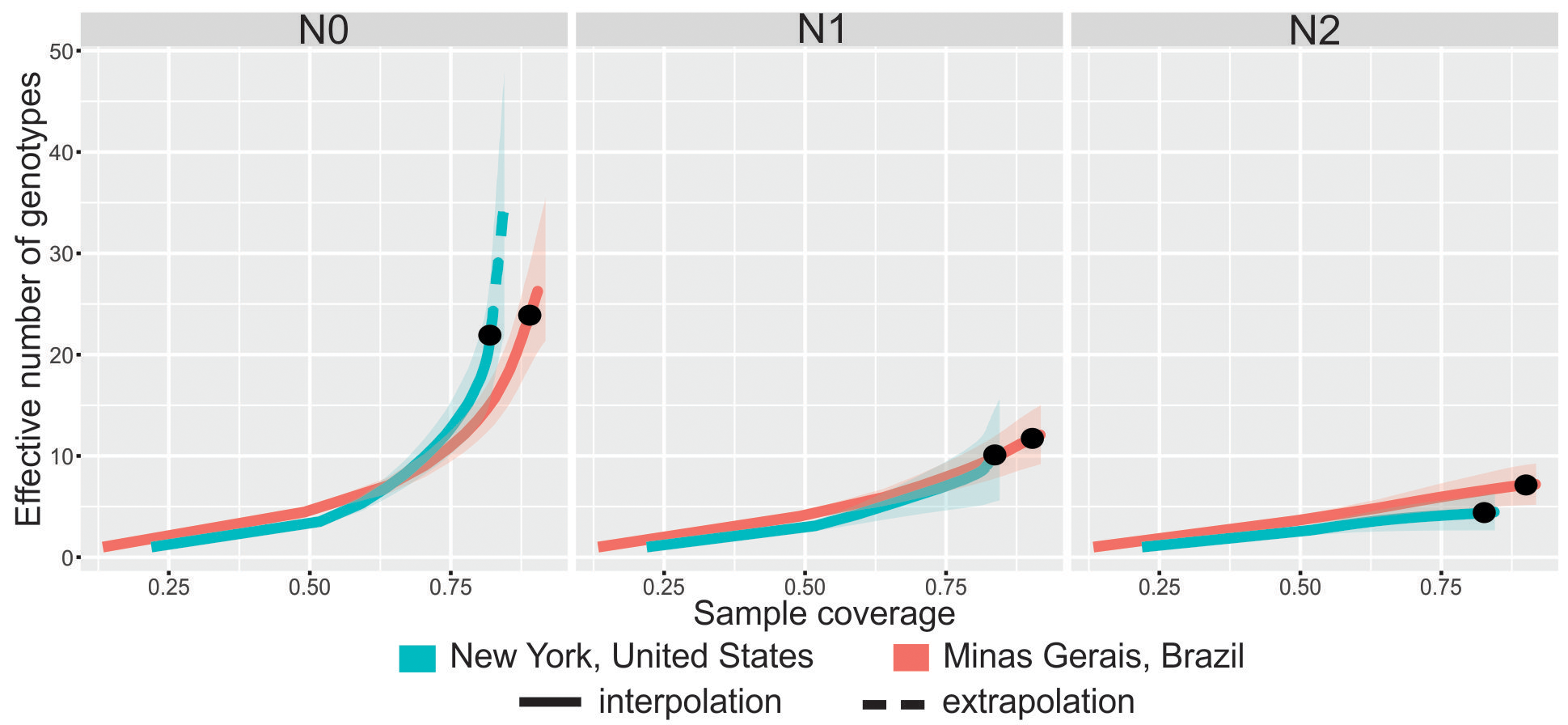

Supplement: S2 Fig — The N0, N1 and N2 numbers correspond to genotype richness, the exponential of Shannon’s entropy, and the inverse of the Simpson’s concentration indices, respectively. Solid lines correspond to rarefaction (interpolation) and dashed lines to extrapolation curves. The 95% confidence intervals were obtained by a bootstrap method based on 200 replications. (TIF) [file pone.0173915.s002.tif]
