# Supplementary material for: Independently founded populations of Sclerotinia sclerotiorum from a tropical and a temperate region have similar genetic structure
Source: PLoS One. 2017 Mar 15;12(3):e0173915. doi: 10.1371/journal.pone.0173915 (PMC5352009; doi:10.1371/journal.pone.0173915)
Supplement: S1 Table — (DOCX) [file pone.0173915.s003.docx]

S1 Table. Mycelial compatibility group (MCG) and allele size at each microsatellite locus of the *Sclerotinia sclerotiorum* isolates from New York, United States of America (USA), and Minas Gerais, Brazil (BRA).

| Isolate code/Origin | MCG | Microsatellite allele size | | | | | | | | | | | | |
| --- | --- | --- | --- | --- | --- | --- | --- | --- | --- | --- | --- | --- | --- | --- |
|  |  | Locus | | | | | | | | | | | | |
|  |  | 55-4 | 13-2 | 110-4 | 36-4 | 42-4 | 8-3 | 5-2 | 17-3 | 114-4 | 7-2 | 12-2 | 9-2 | 92-4 |
| **New York (USA)** |  |  |  |  |  |  |  |  |  |  |  |  |  |  |
| 14115-24 | USA-5 | 165 | 310 | 384 | 411 | 406 | 250 | 319 | 347 | 358 | 172 | 217 | 358 | 376 |
| 14116-11 | USA-1 | 173 | 310 | 376 | 411 | 406 | 250 | 319 | 347 | 366 | 172 | 223 | 358 | 376 |
| 14119-8 | USA-8 | 169 | 310 | 368 | 411 | 406 | 250 | 317 | 341 | 354 | 170 | 219 | 358 | 372 |
| 14120 | USA-1 | 173 | 310 | 376 | 411 | 406 | 250 | 319 | 347 | 366 | 172 | 223 | 358 | 376 |
| 14121 | USA-5 | 165 | 310 | 384 | 411 | 406 | 250 | 319 | 347 | 358 | 172 | 217 | 358 | 376 |
| 14122-5 | USA-1 | 173 | 310 | 376 | 411 | 406 | 250 | 319 | 347 | 366 | 172 | 223 | 358 | 376 |
| 14123-29 | USA-1 | 173 | 310 | 376 | 411 | 406 | 250 | 319 | 347 | 366 | 172 | 223 | 358 | 376 |
| 14124-5 | USA-1 | 173 | 310 | 376 | 411 | 406 | 250 | 319 | 347 | 366 | 172 | 223 | 358 | 376 |
| 14125-8 | USA-7 | 165 | 310 | 384 | 411 | 406 | 250 | 319 | 347 | 366 | 172 | 217 | 360 | 376 |
| 14126-7 | USA-5 | 173 | 310 | 376 | 411 | 406 | 250 | 319 | 353 | 362 | 172 | 217 | 358 | 376 |
| 14127-11 | USA-2 | 173 | 300 | 376 | 411 | 406 | 252 | 317 | 347 | 354 | 172 | 219 | 358 | 374 |
| 14129-13 | USA-1 | 173 | 310 | 376 | 411 | 406 | 250 | 319 | 347 | 366 | 172 | 223 | 358 | 376 |
| 14132-20 | USA-3 | 173 | 300 | 376 | 411 | 406 | 252 | 317 | 347 | 370 | 172 | 217 | 358 | 374 |
| 14133-10 | USA-10 | 165 | 310 | 368 | 411 | 406 | 252 | 317 | 347 | 370 | 172 | 219 | 358 | 372 |
| 14134 | USA-1 | 173 | 310 | 376 | 411 | 406 | 250 | 319 | 347 | 366 | 172 | 223 | 358 | 376 |
| 14136-8 | USA-4 | 173 | 300 | 376 | 411 | 406 | 252 | 317 | 347 | 370 | 172 | 217 | 358 | 372 |
| 14137 | USA-4 | 173 | 300 | 376 | 411 | 406 | 252 | 317 | 347 | 370 | 172 | 217 | 358 | 372 |
| 14138-9 | USA-1 | 173 | 310 | 376 | 411 | 406 | 250 | 319 | 347 | 366 | 172 | 223 | 358 | 376 |
| 14139 | USA-18 | 165 | 310 | 368 | 411 | 406 | 252 | 317 | 347 | 370 | 172 | 219 | 358 | 372 |
| 14140 | USA-1 | 173 | 310 | 376 | 411 | 406 | 250 | 319 | 347 | 366 | 172 | 223 | 358 | 376 |
| 14141-1 | USA-1 | 173 | 310 | 376 | 411 | 406 | 250 | 319 | 347 | 366 | 172 | 223 | 358 | 376 |
| 14142 | USA-19 | 173 | 300 | 376 | 411 | 406 | 252 | 317 | 347 | 370 | 172 | 217 | 358 | 374 |
| 14144 | USA-1 | 173 | 310 | 376 | 411 | 406 | 250 | 319 | 347 | 366 | 172 | 223 | 358 | 376 |
| 14146-10 | USA-1 | 173 | 310 | 376 | 411 | 406 | 250 | 319 | 347 | 366 | 172 | 223 | 358 | 376 |
| 14147 | USA-1 | 173 | 310 | 376 | 411 | 406 | 250 | 319 | 347 | 366 | 172 | 223 | 358 | 376 |
| 14148 | USA-20 | 165 | 310 | 384 | 411 | 406 | 250 | 319 | 347 | 366 | 172 | 217 | 360 | 376 |
| 14149-11 | USA-5 | 165 | 310 | 384 | 411 | 406 | 250 | 319 | 347 | 358 | 172 | 217 | 358 | 376 |
| 14150 | USA-1 | 173 | 310 | 376 | 411 | 406 | 250 | 319 | 347 | 366 | 172 | 223 | 358 | 376 |
| 14151-7 | USA-5 | 165 | 310 | 384 | 411 | 406 | 250 | 319 | 347 | 358 | 172 | 217 | 358 | 376 |
| 14152 | USA-3 | 173 | 300 | 376 | 411 | 406 | 252 | 317 | 353 | 370 | 172 | 217 | 358 | 374 |
| 14153-28 | USA-1 | 173 | 310 | 376 | 411 | 406 | 250 | 319 | 347 | 366 | 172 | 223 | 358 | 376 |
| 14155 | USA-8 | 169 | 310 | 368 | 411 | 406 | 252 | 317 | 353 | 354 | 170 | 219 | 358 | 372 |
| 14156-29 | USA-1 | 173 | 310 | 376 | 411 | 406 | 250 | 319 | 347 | 366 | 172 | 223 | 358 | 376 |
| 14157 | USA-5 | 165 | 310 | 384 | 411 | 406 | 250 | 319 | 347 | 358 | 172 | 217 | 358 | 376 |
| 14158-10 | USA-5 | 165 | 310 | 384 | 411 | 406 | 250 | 319 | 347 | 358 | 172 | 217 | 358 | 376 |
| 14159 | USA-1 | 173 | 310 | 376 | 411 | 406 | 250 | 319 | 347 | 366 | 172 | 223 | 358 | 376 |
| 14160 | USA-1 | 173 | 310 | 376 | 411 | 406 | 248 | 319 | 347 | 366 | 172 | 223 | 358 | 376 |
| 14161 | USA-1 | 173 | 310 | 376 | 411 | 406 | 250 | 319 | 347 | 366 | 172 | 223 | 358 | 376 |
| 14174-14 | USA-6 | 157 | 300 | 368 | 411 | 406 | 250 | 317 | 347 | 358 | 170 | 219 | 358 | 372 |
| 14175 | USA-8 | 169 | 310 | 368 | 411 | 406 | 252 | 317 | 341 | 354 | 170 | 219 | 358 | 372 |
| 14176 | USA-3 | 173 | 300 | 376 | 411 | 406 | 252 | 317 | 347 | 370 | 172 | 217 | 358 | 374 |
| 14177 | USA-1 | 173 | 310 | 376 | 411 | 406 | 250 | 319 | 347 | 366 | 172 | 223 | 358 | 376 |
| 14178 | USA-1 | 173 | 310 | 376 | 411 | 406 | 250 | 319 | 347 | 366 | 172 | 223 | 358 | 376 |
| 14179-3 | USA-16 | 173 | 310 | 376 | 411 | 406 | 250 | 319 | 347 | 366 | 172 | 223 | 358 | 376 |
| 14204 | USA-12 | 157 | 350 | 368 | 411 | 406 | 250 | 317 | 347 | 370 | 170 | 223 | 358 | 372 |
| 14205 | USA-13 | 157 | 300 | 368 | 411 | 406 | 254 | 317 | 347 | 370 | 174 | 223 | 358 | 372 |
| 14206-12 | USA-7 | 165 | 310 | 384 | 411 | 406 | 250 | 319 | 347 | 366 | 172 | 217 | 360 | 376 |
| 14207-6 | USA-3 | 173 | 300 | 376 | 411 | 406 | 250 | 317 | 347 | 370 | 172 | 217 | 358 | 374 |
| 14208 | USA-1 | 173 | 310 | 376 | 411 | 406 | 250 | 319 | 347 | 366 | 172 | 223 | 358 | 376 |
| 14209 | USA-3 | 173 | 300 | 376 | 411 | 406 | 252 | 317 | 347 | 370 | 172 | 217 | 358 | 374 |
| 14210-9 | USA-1 | 173 | 310 | 376 | 411 | 406 | 250 | 319 | 347 | 366 | 172 | 223 | 358 | 376 |
| 14211 | USA-13 | 173 | 300 | 376 | 411 | 406 | 252 | 317 | 347 | 370 | 172 | 217 | 358 | 372 |
| 14212-23 | USA-5 | 165 | 310 | 384 | 411 | 406 | 250 | 319 | 347 | 358 | 172 | 217 | 358 | 376 |
| 14213 | USA-1 | 173 | 310 | 376 | 411 | 406 | 250 | 319 | 347 | 366 | 172 | 223 | 358 | 376 |
| 14214 | USA-1 | 173 | 310 | 376 | 411 | 406 | 250 | 319 | 347 | 366 | 172 | 223 | 358 | 376 |
| 14215 | USA-1 | 173 | 310 | 376 | 411 | 406 | 250 | 319 | 347 | 366 | 172 | 223 | 358 | 376 |
| 14217 | USA-5 | 165 | 310 | 384 | 411 | 406 | 250 | 319 | 347 | 358 | 172 | 217 | 358 | 376 |
| 14218 | USA-1 | 173 | 310 | 376 | 411 | 406 | 250 | 319 | 347 | 366 | 172 | 223 | 358 | 376 |
| 14219-17 | USA-4 | 173 | 300 | 376 | 411 | 406 | 252 | 317 | 347 | 370 | 172 | 217 | 358 | 372 |
| 14220 | USA-1 | 173 | 310 | 376 | 411 | 406 | 250 | 319 | 347 | 366 | 172 | 223 | 358 | 376 |
| 14221-3 | USA-1 | 173 | 310 | 376 | 411 | 406 | 250 | 319 | 347 | 366 | 172 | 223 | 358 | 376 |
| 14222-7 | USA-11 | 173 | 300 | 368 | 411 | 406 | 252 | 317 | 347 | 370 | 174 | 223 | 358 | 372 |
| 14223 | USA-1 | 173 | 310 | 376 | 411 | 406 | 250 | 319 | 347 | 366 | 172 | 223 | 358 | 376 |
| 14224 | USA-1 | 173 | 310 | 384 | 411 | 406 | 250 | 319 | 347 | 366 | 172 | 217 | 358 | 376 |
| 14225-14 | USA-1 | 173 | 310 | 376 | 411 | 406 | 250 | 319 | 347 | 366 | 172 | 223 | 358 | 376 |
| 14226 | USA-3 | 173 | 300 | 376 | 411 | 406 | 252 | 317 | 347 | 370 | 172 | 217 | 358 | 374 |
| 14227 | USA-1 | 173 | 310 | 376 | 411 | 406 | 250 | 319 | 347 | 366 | 172 | 223 | 358 | 376 |
| 14241 | USA-8 | 169 | 310 | 368 | 411 | 406 | 252 | 317 | 341 | 354 | 170 | 219 | 358 | 372 |
| 14242 | USA-14 | 173 | 310 | 384 | 411 | 406 | 250 | 319 | 347 | 358 | 172 | 217 | 358 | 376 |
| 14243 | USA-1 | 173 | 310 | 376 | 411 | 406 | 250 | 319 | 347 | 366 | 172 | 223 | 358 | 376 |
| 14244-3 | USA-9 | 169 | 300 | 368 | 411 | 406 | 250 | 317 | 341 | 354 | 172 | 223 | 358 | 372 |
| 14245 | USA-5 | 165 | 310 | 384 | 411 | 406 | 250 | 319 | 347 | 358 | 172 | 217 | 358 | 376 |
| 14246-3 | USA-8 | 169 | 310 | 368 | 411 | 406 | 250 | 317 | 341 | 354 | 170 | 219 | 358 | 372 |
| 14247 | USA-1 | 173 | 310 | 376 | 411 | 406 | 250 | 319 | 347 | 366 | 172 | 223 | 358 | 376 |
| 14248 | USA-15 | 169 | 310 | 368 | 411 | 406 | 250 | 317 | 341 | 354 | 170 | 219 | 358 | 372 |
| 14249 | USA-21 | 165 | 310 | 368 | 411 | 406 | 252 | 317 | 341 | 354 | 170 | 219 | 358 | 372 |
| 14250 | USA-1 | 173 | 310 | 376 | 411 | 406 | 250 | 319 | 347 | 366 | 172 | 223 | 358 | 376 |
| 14251-9 | USA-8 | 169 | 310 | 368 | 411 | 406 | 252 | 317 | 341 | 354 | 170 | 219 | 358 | 372 |
| **Minas Gerais (Brazil)*** |  |  |  |  |  |  |  |  |  |  |  |  |  |  |
| Ss-1 | BRA-1 | 157 | 305 | 372 | 411 | 406 | 252 | 317 | 359 | 410 | 162 | 219 | 358 | 374 |
| Ss-2 | BRA-1 | 157 | 305 | 372 | 411 | 406 | 252 | 317 | 359 | 410 | 162 | 219 | 358 | 374 |
| Ss-4 | BRA-12 | 157 | 290 | 372 | 411 | 406 | 252 | 315 | 359 | 358 | 170 | 223 | 358 | 374 |
| Ss-5 | BRA-2 | 177 | 305 | 368 | 411 | 406 | 250 | 317 | 347 | 410 | 174 | 223 | 358 | 376 |
| Ss-7 | BRA-2 | 157 | 305 | 372 | 411 | 406 | 252 | 317 | 359 | 410 | 162 | 219 | 358 | 374 |
| Ss-8 | BRA-1 | 157 | 305 | 372 | 411 | 406 | 250 | 317 | 359 | 410 | 162 | 219 | 358 | 374 |
| Ss-9 | BRA-1 | 157 | 305 | 372 | 411 | 406 | 252 | 317 | 359 | 410 | 162 | 219 | 358 | 374 |
| Ss-10 | BRA-1 | 157 | 305 | 372 | 411 | 406 | 250 | 317 | 359 | 410 | 162 | 219 | 358 | 374 |
| Ss-11 | BRA-1 | 157 | 305 | 372 | 411 | 406 | 250 | 317 | 359 | 410 | 162 | 219 | 358 | 374 |
| Ss-14 | BRA-1 | 157 | 305 | 372 | 411 | 406 | 252 | 317 | 359 | 414 | 162 | 219 | 358 | 374 |
| Ss-15 | BRA-6 | 157 | 290 | 368 | 411 | 406 | 250 | 319 | 356 | 358 | 170 | 223 | 358 | 374 |
| Ss-18 | BRA-8 | 157 | 290 | 372 | 411 | 406 | 250 | 315 | 353 | 362 | 170 | 224 | 358 | 374 |
| Ss-19 | BRA-1 | 157 | 305 | 372 | 411 | 406 | 252 | 317 | 359 | 410 | 162 | 219 | 358 | 374 |
| Ss-22 | BRA-6 | 157 | 290 | 368 | 411 | 406 | 250 | 319 | 356 | 358 | 170 | 223 | 358 | 374 |
| Ss-23 | BRA-1 | 157 | 305 | 372 | 411 | 406 | 252 | 317 | 359 | 410 | 162 | 219 | 358 | 374 |
| Ss-26 | BRA-1 | 157 | 305 | 372 | 411 | 406 | 252 | 317 | 359 | 410 | 162 | 219 | 358 | 374 |
| Ss-27 | BRA-1 | 157 | 305 | 372 | 411 | 406 | 252 | 317 | 359 | 410 | 162 | 219 | 358 | 374 |
| Ss-28 | BRA-1 | 157 | 305 | 372 | 411 | 406 | 250 | 317 | 359 | 410 | 162 | 219 | 358 | 374 |
| Ss-29 | BRA-1 | 157 | 305 | 372 | 411 | 406 | 250 | 317 | 359 | 410 | 162 | 219 | 358 | 374 |
| Ss-30 | BRA-1 | 157 | 305 | 372 | 411 | 406 | 250 | 317 | 359 | 410 | 162 | 219 | 358 | 374 |
| Ss-31 | BRA-1 | 157 | 305 | 372 | 411 | 406 | 250 | 317 | 359 | 410 | 162 | 219 | 358 | 374 |
| Ss-32 | BRA-1 | 157 | 305 | 372 | 411 | 406 | 252 | 317 | 359 | 410 | 162 | 219 | 358 | 374 |
| Ss-33 | BRA-6 | 157 | 290 | 368 | 411 | 406 | 254 | 319 | 356 | 358 | 170 | 223 | 358 | 374 |
| Ss-35 | BRA-1 | 157 | 305 | 372 | 411 | 406 | 250 | 317 | 359 | 410 | 162 | 219 | 358 | 374 |
| Ss-36 | BRA-1 | 157 | 305 | 372 | 411 | 406 | 252 | 317 | 359 | 410 | 162 | 219 | 358 | 374 |
| Ss-37 | BRA-1 | 157 | 305 | 372 | 411 | 406 | 252 | 317 | 359 | 410 | 162 | 219 | 358 | 374 |
| Ss-38 | BRA-1 | 157 | 305 | 372 | 411 | 406 | 250 | 317 | 359 | 410 | 162 | 219 | 358 | 374 |
| Ss-39 | BRA-1 | 157 | 305 | 372 | 411 | 406 | 252 | 317 | 359 | 410 | 162 | 219 | 358 | 374 |
| Ss-41 | BRA-1 | 157 | 305 | 372 | 411 | 406 | 250 | 317 | 359 | 410 | 162 | 219 | 358 | 374 |
| Ss-42 | BRA-8 | 157 | 290 | 372 | 411 | 406 | 254 | 315 | 353 | 362 | 170 | 223 | 358 | 374 |
| Ss-43 | BRA-1 | 157 | 305 | 372 | 411 | 406 | 252 | 317 | 359 | 410 | 162 | 219 | 358 | 374 |
| Ss-44 | BRA-4 | 189 | 310 | 372 | 411 | 406 | 244 | 317 | 353 | 338 | 160 | 217 | 358 | 372 |
| Ss-46 | BRA-1 | 157 | 305 | 372 | 411 | 406 | 250 | 317 | 359 | 410 | 162 | 219 | 358 | 374 |
| Ss-47 | BRA-1 | 157 | 305 | 372 | 411 | 406 | 250 | 317 | 359 | 410 | 162 | 219 | 358 | 374 |
| Ss-48 | BRA-2 | 177 | 305 | 368 | 411 | 406 | 252 | 317 | 347 | 410 | 174 | 223 | 358 | 376 |
| Ss-49 | BRA-1 | 157 | 305 | 372 | 411 | 406 | 250 | 317 | 359 | 410 | 162 | 219 | 358 | 374 |
| Ss-50 | BRA-7 | 157 | 320 | 368 | 411 | 406 | 244 | 317 | 353 | 338 | 160 | 217 | 360 | 372 |
| Ss-51 | BRA-4 | 189 | 310 | 372 | 411 | 406 | 244 | 317 | 353 | 338 | 160 | 217 | 358 | 372 |
| Ss-52 | BRA-1 | 157 | 305 | 372 | 411 | 406 | 250 | 317 | 359 | 410 | 162 | 219 | 358 | 374 |
| Ss-53 | BRA-2 | 177 | 305 | 368 | 411 | 406 | 250 | 317 | 347 | 410 | 174 | 223 | 358 | 376 |
| Ss-54 | BRA-1 | 157 | 305 | 372 | 411 | 406 | 250 | 317 | 359 | 410 | 162 | 219 | 358 | 374 |
| Ss-56 | BRA-4 | 189 | 310 | 372 | 411 | 406 | 244 | 317 | 353 | 338 | 160 | 217 | 358 | 372 |
| Ss-57 | BRA-1 | 157 | 305 | 372 | 411 | 406 | 250 | 317 | 359 | 410 | 162 | 219 | 358 | 374 |
| Ss-58 | BRA-2 | 177 | 305 | 368 | 411 | 406 | 252 | 317 | 347 | 410 | 174 | 223 | 358 | 376 |
| Ss-59 | BRA-13 | 157 | 275 | 368 | 411 | 406 | 250 | 315 | 374 | 370 | 170 | 223 | 358 | 378 |
| Ss-60 | BRA-2 | 177 | 305 | 368 | 411 | 406 | 250 | 317 | 347 | 410 | 170 | 223 | 358 | 376 |
| Ss-61 | BRA-1 | 157 | 305 | 372 | 411 | 406 | 250 | 317 | 359 | 410 | 162 | 219 | 358 | 374 |
| Ss-62 | BRA-2 | 177 | 305 | 368 | 411 | 406 | 250 | 317 | 347 | 410 | 174 | 223 | 358 | 376 |
| Ss-63 | BRA-2 | 177 | 305 | 368 | 411 | 406 | 250 | 317 | 347 | 410 | 174 | 223 | 358 | 376 |
| Ss-64 | BRA-2 | 177 | 305 | 368 | 411 | 406 | 252 | 317 | 347 | 410 | 174 | 223 | 358 | 376 |
| Ss-65 | BRA-2 | 177 | 305 | 368 | 411 | 406 | 250 | 317 | 347 | 410 | 174 | 223 | 358 | 376 |
| Ss-66 | BRA-4 | 189 | 310 | 372 | 411 | 406 | 244 | 317 | 353 | 338 | 160 | 217 | 358 | 372 |
| Ss-67 | BRA-1 | 157 | 305 | 372 | 411 | 406 | 252 | 317 | 359 | 414 | 162 | 219 | 358 | 374 |
| Ss-68 | BRA-2 | 177 | 305 | 368 | 411 | 406 | 252 | 317 | 347 | 410 | 174 | 223 | 358 | 376 |
| Ss-69 | BRA-14 | 157 | 290 | 372 | 411 | 406 | 256 | 317 | 350 | 354 | 174 | 217 | 370 | 374 |
| Ss-70 | BRA-2 | 177 | 305 | 368 | 411 | 406 | 252 | 317 | 347 | 414 | 174 | 223 | 358 | 376 |
| Ss-71 | BRA-2 | 177 | 305 | 368 | 411 | 406 | 252 | 317 | 347 | 410 | 174 | 223 | 358 | 376 |
| Ss-72 | BRA-4 | 189 | 310 | 372 | 411 | 406 | 244 | 317 | 353 | 338 | 160 | 217 | 358 | 372 |
| Ss-77 | BRA-1 | 157 | 305 | 372 | 411 | 406 | 250 | 317 | 359 | 410 | 162 | 219 | 358 | 374 |
| Ss-78 | BRA-1 | 157 | 305 | 372 | 411 | 406 | 250 | 317 | 359 | 410 | 162 | 219 | 358 | 374 |
| Ss-79 | BRA-2 | 177 | 305 | 368 | 411 | 406 | 250 | 317 | 347 | 410 | 174 | 223 | 358 | 376 |
| Ss-81 | BRA-5 | 157 | 275 | 372 | 411 | 406 | 250 | 315 | 341 | 386 | 170 | 215 | 358 | 378 |
| Ss-83 | BRA-10 | 153 | 275 | 368 | 411 | 406 | 268 | 317 | 374 | 386 | 170 | 215 | 358 | 372 |
| Ss-85 | BRA-3 | 157 | 275 | 368 | 411 | 406 | 250 | 315 | 374 | 370 | 170 | 223 | 358 | 378 |
| Ss-86 | BRA-3 | 157 | 275 | 368 | 411 | 406 | 250 | 315 | 374 | 370 | 170 | 223 | 358 | 378 |
| Ss-89 | BRA-3 | 157 | 275 | 368 | 411 | 406 | 250 | 315 | 374 | 370 | 170 | 223 | 358 | 378 |
| Ss-90 | BRA-5 | 157 | 275 | 372 | 411 | 406 | 250 | 315 | 341 | 386 | 170 | 215 | 358 | 378 |
| Ss-91 | BRA-10 | 153 | 275 | 368 | 411 | 406 | 268 | 317 | 374 | 386 | 170 | 215 | 358 | 372 |
| Ss-92 | BRA-5 | 157 | 275 | 372 | 411 | 406 | 252 | 315 | 341 | 386 | 170 | 215 | 358 | 378 |
| Ss-93 | BRA-3 | 157 | 275 | 368 | 411 | 406 | 250 | 315 | 374 | 370 | 170 | 223 | 358 | 378 |
| Ss-94 | BRA-3 | 157 | 275 | 368 | 411 | 406 | 250 | 315 | 374 | 370 | 170 | 223 | 358 | 378 |
| Ss-95 | BRA-3 | 157 | 275 | 368 | 411 | 406 | 250 | 315 | 374 | 370 | 170 | 223 | 358 | 378 |
| Ss-96 | BRA-2 | 177 | 305 | 368 | 411 | 406 | 250 | 317 | 347 | 410 | 174 | 223 | 358 | 376 |
| Ss-97 | BRA-3 | 157 | 275 | 368 | 411 | 406 | 250 | 315 | 374 | 370 | 170 | 223 | 358 | 378 |
| Ss-98 | BRA-3 | 157 | 275 | 368 | 411 | 406 | 250 | 315 | 374 | 370 | 170 | 223 | 358 | 378 |
| Ss-99 | BRA-5 | 157 | 275 | 372 | 411 | 406 | 250 | 315 | 341 | 386 | 170 | 215 | 358 | 378 |
| Ss-100 | BRA-3 | 157 | 275 | 368 | 411 | 406 | 250 | 315 | 374 | 370 | 170 | 223 | 358 | 378 |
| Ss-101 | BRA-1 | 157 | 305 | 372 | 411 | 406 | 252 | 317 | 359 | 410 | 162 | 219 | 358 | 374 |
| Ss-102 | BRA-11 | 197 | 275 | 368 | 411 | 406 | 268 | 315 | 374 | 370 | 170 | 223 | 358 | 378 |
| Ss-103 | BRA-1 | 157 | 305 | 372 | 411 | 406 | 250 | 317 | 359 | 410 | 162 | 219 | 358 | 374 |
| Ss-104 | BRA-1 | 157 | 305 | 372 | 411 | 406 | 250 | 317 | 359 | 410 | 162 | 219 | 358 | 374 |
| Ss-105 | BRA-1 | 157 | 305 | 372 | 411 | 406 | 250 | 317 | 359 | 410 | 162 | 219 | 358 | 374 |
| Ss-106 | BRA-1 | 157 | 305 | 372 | 411 | 406 | 252 | 317 | 359 | 410 | 162 | 219 | 358 | 374 |
| Ss-107 | BRA-1 | 157 | 305 | 372 | 411 | 406 | 250 | 317 | 359 | 410 | 162 | 219 | 358 | 374 |
| Ss-109 | BRA-7 | 157 | 320 | 368 | 411 | 406 | 244 | 317 | 353 | 338 | 160 | 217 | 360 | 372 |
| Ss-110 | BRA-7 | 157 | 320 | 368 | 411 | 406 | 244 | 317 | 353 | 338 | 160 | 217 | 360 | 372 |
| Ss-115 | BRA-7 | 157 | 320 | 368 | 411 | 406 | 244 | 317 | 353 | 338 | 160 | 217 | 360 | 372 |
| Ss-116 | BRA-2 | 177 | 305 | 368 | 411 | 406 | 250 | 317 | 347 | 414 | 174 | 223 | 358 | 376 |
| Ss-118 | BRA-1 | 157 | 305 | 372 | 411 | 406 | 252 | 317 | 359 | 410 | 162 | 219 | 358 | 374 |
| Ss-119 | BRA-1 | 157 | 305 | 372 | 411 | 406 | 252 | 317 | 359 | 410 | 162 | 219 | 358 | 374 |
| Ss-120 | BRA-7 | 157 | 320 | 368 | 411 | 406 | 244 | 317 | 353 | 338 | 160 | 217 | 360 | 372 |
| Ss-121 | BRA-2 | 177 | 305 | 368 | 411 | 406 | 252 | 317 | 347 | 410 | 174 | 223 | 358 | 376 |
| Ss-122 | BRA-1 | 157 | 305 | 372 | 411 | 406 | 252 | 317 | 359 | 410 | 162 | 219 | 358 | 374 |
| Ss-123 | BRA-9 | 157 | 290 | 368 | 411 | 406 | 256 | 317 | 359 | 414 | 162 | 223 | 358 | 374 |
| Ss-125 | BRA-9 | 157 | 290 | 368 | 411 | 406 | 256 | 317 | 359 | 414 | 162 | 223 | 358 | 374 |
| Ss-126 | BRA-2 | 177 | 305 | 368 | 411 | 406 | 250 | 317 | 347 | 402 | 176 | 223 | 358 | 376 |
| Ss-127 | BRA-1 | 157 | 305 | 372 | 411 | 406 | 252 | 317 | 359 | 410 | 162 | 219 | 358 | 374 |
| Ss-128 | BRA-1 | 157 | 305 | 372 | 411 | 406 | 250 | 317 | 359 | 410 | 162 | 219 | 358 | 374 |
| Ss-130 | BRA-1 | 157 | 305 | 372 | 411 | 406 | 250 | 317 | 359 | 410 | 162 | 219 | 358 | 374 |
| Ss-131 | BRA-1 | 157 | 305 | 372 | 411 | 406 | 252 | 317 | 359 | 410 | 162 | 219 | 358 | 374 |
| Ss-246 | BRA-1 | 157 | 305 | 372 | 411 | 406 | 252 | 317 | 359 | 410 | 162 | 219 | 358 | 374 |
| Ss-247 | BRA-1 | 157 | 305 | 372 | 411 | 406 | 252 | 317 | 359 | 410 | 162 | 219 | 358 | 374 |
| Ss-248 | BRA-1 | 157 | 305 | 372 | 411 | 406 | 252 | 317 | 359 | 410 | 162 | 219 | 358 | 374 |
| Ss-249 | BRA-1 | 157 | 305 | 372 | 411 | 406 | 252 | 317 | 359 | 410 | 162 | 219 | 358 | 374 |
| Ss-250 | BRA-1 | 157 | 305 | 372 | 411 | 406 | 250 | 317 | 359 | 410 | 162 | 219 | 358 | 374 |
| Ss-251 | BRA-1 | 157 | 305 | 372 | 411 | 406 | 252 | 317 | 359 | 410 | 162 | 219 | 358 | 374 |
| Ss-252 | BRA-1 | 157 | 305 | 372 | 411 | 406 | 252 | 317 | 359 | 410 | 162 | 219 | 358 | 374 |
| Ss-258 | BRA-1 | 157 | 305 | 372 | 411 | 406 | 250 | 317 | 359 | 410 | 162 | 219 | 358 | 374 |
| Ss-260 | BRA-1 | 157 | 305 | 372 | 411 | 406 | 252 | 317 | 359 | 410 | 162 | 217 | 358 | 374 |

* The Brazilian isolates were previously genotyped at 10 loci (Lehner et al., 2015) and re-genotyped in the current study at 13 loci. Slight modifications in allele size were observed, but overall the allelic composition was highly similar.
